# Learning to Make Rare and Complex Diagnoses With Generative AI Assistance: Qualitative Study of Popular Large Language Models

**DOI:** 10.2196/51391

**Published:** 2024-02-13

**Authors:** Tassallah Abdullahi, Ritambhara Singh, Carsten Eickhoff

**Affiliations:** 1 Department of Computer Science Brown University Providence, RI United States; 2 Center for Computational Molecular Biology Brown University Providence, RI United States; 3 School of Medicine University of Tübingen Tübingen Germany

**Keywords:** clinical decision support, rare diseases, complex diseases, prompt engineering, reliability, consistency, natural language processing, language model, Bard, ChatGPT 3.5, GPT-4, MedAlpaca, medical education, complex diagnosis, artificial intelligence, AI assistance, medical training, prediction model

## Abstract

**Background:**

Patients with rare and complex diseases often experience delayed diagnoses and misdiagnoses because comprehensive knowledge about these diseases is limited to only a few medical experts. In this context, large language models (LLMs) have emerged as powerful knowledge aggregation tools with applications in clinical decision support and education domains.

**Objective:**

This study aims to explore the potential of 3 popular LLMs, namely Bard (Google LLC), ChatGPT-3.5 (OpenAI), and GPT-4 (OpenAI), in medical education to enhance the diagnosis of rare and complex diseases while investigating the impact of prompt engineering on their performance.

**Methods:**

We conducted experiments on publicly available complex and rare cases to achieve these objectives. We implemented various prompt strategies to evaluate the performance of these models using both open-ended and multiple-choice prompts. In addition, we used a majority voting strategy to leverage diverse reasoning paths within language models, aiming to enhance their reliability. Furthermore, we compared their performance with the performance of human respondents and MedAlpaca, a generative LLM specifically designed for medical tasks.

**Results:**

Notably, all LLMs outperformed the average human consensus and MedAlpaca, with a minimum margin of 5% and 13%, respectively, across all 30 cases from the diagnostic case challenge collection. On the frequently misdiagnosed cases category, Bard tied with MedAlpaca but surpassed the human average consensus by 14%, whereas GPT-4 and ChatGPT-3.5 outperformed MedAlpaca and the human respondents on the moderately often misdiagnosed cases category with minimum accuracy scores of 28% and 11%, respectively. The majority voting strategy, particularly with GPT-4, demonstrated the highest overall score across all cases from the diagnostic complex case collection, surpassing that of other LLMs. On the Medical Information Mart for Intensive Care-III data sets, Bard and GPT-4 achieved the highest diagnostic accuracy scores, with multiple-choice prompts scoring 93%, whereas ChatGPT-3.5 and MedAlpaca scored 73% and 47%, respectively. Furthermore, our results demonstrate that there is no one-size-fits-all prompting approach for improving the performance of LLMs and that a single strategy does not universally apply to all LLMs.

**Conclusions:**

Our findings shed light on the diagnostic capabilities of LLMs and the challenges associated with identifying an optimal prompting strategy that aligns with each language model’s characteristics and specific task requirements. The significance of prompt engineering is highlighted, providing valuable insights for researchers and practitioners who use these language models for medical training. Furthermore, this study represents a crucial step toward understanding how LLMs can enhance diagnostic reasoning in rare and complex medical cases, paving the way for developing effective educational tools and accurate diagnostic aids to improve patient care and outcomes.

## Introduction

### Background

Natural language processing has witnessed remarkable advances with the introduction of generative large language models (LLMs). In November 2022, OpenAI released ChatGPT-3.5 (OpenAI), a large natural language processing chatbot trained on a large corpus collected from the internet to generate humanlike text in response to user queries. ChatGPT-3.5 has seen massive popularity, and users have praised its creativity and language comprehension for several tasks, such as text summarization and writing computer programs [1]. In March 2023, OpenAI responded to the success of ChatGPT-3.5 by introducing an enhanced iteration called GPT-4, specifically designed to address intricate queries and nuanced directives more effectively. Shortly thereafter, Google released their comparable model, Bard (Google LLC), which joined the league of impressive LLMs. What sets Bard apart is its real-time access to and use of internet information, enriching its response generation with up-to-date information [2]. In contrast, GPT-4 possesses multimodal capabilities, including image inputs, albeit not publicly available during the study [3].

These LLMs were not originally designed for medical applications. However, several studies [4,5] have shown their extraordinary capabilities in excelling in various medical examinations, such as the Self-Assessment in Neurological Surgery examination and the USMLE (United States Medical Licensing Examination). Their results demonstrated the ability of these models to handle clinical information and complex counterfactuals. Furthermore, numerous investigations [6-8] have revealed the remarkable advantages of harnessing the power of LLMs in diverse medical scenarios. Notably, Lee et al [8] demonstrated using LLMs as a reliable conversational agent to collect patient information to assist in medical notetaking, whereas Patel and Lam [9] delved into using LLMs as a valuable tool for generating comprehensive patient discharge summaries. The ability of LLMs to process and generate medical text has unlocked new opportunities to enhance diagnostic reasoning, particularly in tackling rare and complex medical cases.

Rare diseases are characterized by their low prevalence in the general population, whereas complex diseases are conditions with overlapping factors and multiple comorbidities that are often difficult to diagnose [10,11]. Sometimes, a condition can be rare and complex if it is infrequent and challenging to diagnose accurately [11]. Rare and complex diagnoses present significant challenges across various medical levels and often require extensive medical knowledge or expertise for accurate diagnosis and management [10,11]. This may be because, during their education, physicians are trained to prioritize ruling out common diagnoses before considering rare ones during patient evaluation [12]. In addition, most medical education programs rarely cover some complex conditions, and guidance for practicing clinicians is often outdated and inappropriate [13,14]. As a result, most physicians perceive their knowledge of rare diseases as insufficient or very poor, and only a few feel adequately prepared to care for patients with these conditions [12,15]. This knowledge gap increases the risk of misdiagnosis among individuals with rare and complex conditions. Furthermore, the scarcity of available data and the relatively small number of affected individuals create a complicated diagnostic landscape, even for experienced and specialized clinicians [10]. Consequently, patients often endure a prolonged and arduous diagnostic process. Therefore, there is a pressing need for comprehensive educational tools and accurate diagnostic aids to fill the knowledge gap and address these challenges effectively.

This study aims to explore the potential of 3 LLMs, namely Bard, GPT-4, and ChatGPT-3.5, as continuing medical education (CME) systems to enhance the diagnoses of rare and complex conditions. Although these models have demonstrated impressive success in standardized medical examinations [4,5], it is important to acknowledge that most examinations reflect general clinical situations, which may not fully capture the intricacies encountered in real-world diagnostic scenarios. Furthermore, these standardized tests often feature questions that can be answered through memorization [16]. In contrast, real-world complex diagnostic scenarios that physicians face involve dynamic, multifaceted patient cases with numerous variables and uncertainties. Although previous studies by Liu et al [17] and Cascella et al [18] have highlighted the ability of LLMs to support health care professionals in real-world scenarios, their effectiveness in diagnosing rare and complex conditions remains an area of exploration. Despite the promising use of LLMs in medical applications, studies have reported that their responses to user queries are often nondeterministic (ie, depending on the query format) and exhibit significant variance [17,19]. This attribute may pose challenges in clinical decision support scenarios because the dependability of a system is uncertain when its behavior cannot be accurately predicted. However, no investigation has been conducted to show how different input formats (prompts) affect LLM responses in the medical context.

Prompt engineering is a technique for carefully designing queries (inputs) to improve the performance of generative language models [20,21]. We can guide LLMs to generate more accurate and reliable responses by carefully crafting effective prompts. Our study investigated effective prompting strategies to improve the accuracy and reliability of LLMs in diagnosing rare and complex conditions within an educational context. We evaluated the performance of LLMs by comparing their responses to those of human respondents and the responses of MedAlpaca [22], an open-source generative LLM designed for medical tasks. Given the documented advantages of using LLMs as a complementary tool rather than a substitute for clinicians [17,18], our study incorporated LLMs with the understanding that clinicians may use them beyond real-time diagnostic scenarios. Although our premise is based on a clinician having established an initial diagnostic hypothesis and seeking further assistance to refine the precise diagnosis, we acknowledge the broader utility of LLMs. They can be valuable in real-time decision support and retrospective use during leisure or documentation, allowing physicians to experiment with and enhance their understanding of rare and complex diseases. This approach recognizes the inherent uncertainty in diagnosis and harnesses the capabilities of LLMs to assist clinicians in various aspects of their diagnostic processes. In the context of CME, our study highlights the possibility of integrating LLMs as a valuable addition. By providing further assistance in refining complex and rare diagnoses, these LLMs could support evidence-based decision-making among health care professionals for improved patient outcomes.

### Objectives

Our study has 2 main objectives: first, to examine the potential of LLMs as a CME tool for diagnosing rare and complex conditions, and second, to highlight the impact of prompt formatting on the performance of LLMs. Understanding these aspects could significantly contribute to advancing diagnostic practices and effectively using LLMs to improve patient care.

## Methods

### Data Sets

We used 2 data sets to examine the capacity of LLMs to diagnose rare and complex conditions as follows:

1. Diagnostic case challenge collection (DC3) [11] comprises 30 complex diagnostic cases curated by medical experts in the *New England Journal of Medicine* web-based case challenges. The original cases contained text and image descriptions of patients’ medical history, diagnostic imaging, and laboratory results; however, we used only textual information to form prompts (queries). The web-based polls recorded an average of 5850 (SD 2522.84) respondents per case, many of whom were health care professionals. The participants were required to identify the correct diagnosis from a list of differential diagnoses. Case difficulty was categorized based on the percentage of correct responses received from the respondents on the web-based survey. The case categories were: “rarely misdiagnosed cases” (with ≥21/30, 70% correct responses), “moderately misdiagnosed cases” (with >9/30, 30% and <21/30, 70% correct responses), and “frequently misdiagnosed cases” (with ≤9/30, 30% correct responses). Furthermore, the final diagnoses determined by the treating physicians of the cases were provided alongside the poll results, enabling the comparison of the performance of human respondents with that of the targeted LLMs.
2. Medical Information Mart for Intensive Care-III (MIMIC-III) [23] comprises deidentified electronic health record data from approximately 50,000 Boston Beth Israel Deaconess Medical Center intensive care unit patients. We focused on discharge summaries containing the accumulated patient information from admission to discharge. Similar to previous work on clinical outcome prediction by van Aken et al [24] and Abdullahi et al [25], we filtered document sections unrelated to admissions, such as discharge information or hospital course and retained sections related to admissions, such as chief complaint, history of illness or present illness, medical history, admission medications, allergies, physical examination, family history, and social history. Each discharge summary had a discharge diagnosis section that indicated the patient’s final diagnosis for that admission. We reviewed the discharge summaries to identify rare diseases and referred to the Orphanet website [26]. In this study, we randomly selected 15 unique, rare conditions as our target. These cases were selected as pilot studies for a focused and in-depth analysis.

### Models

In this study, we conducted experiments using LLMs designed for conversational context. Specifically, we used the July 6, 2023, version of Bard; the July 4, 2023, versions of GPT-4 and ChatGPT-3.5; and the publicly available version of MedAlpaca 7b [22]. We entered prompts individually through the chat interface to evaluate Bard, GPT-4, and ChatGPT-3.5, treating each prompt as a distinct conversation. MedAlpaca differs from Bard, ChatGPT-3.5, and GPT-4 in that it requires users to submit queries or prompts through a Python (Python Software Foundation) script. Consequently, we used a single Python script for each prompt strategy to submit queries for each data set. It is worth noting that Bard has certain limitations compared with ChatGPT-3.5 and GPT-4. Bard has a restricted capacity to handle lengthy queries. Moreover, Bard is more sensitive to noisy input and specific characters. For example, the MIMIC-III data set contained deidentified patients’ notes filled with special characters such as “[**Hospital 18654**]” and laboratory results written in shorthand, for example, * Hgb-9.6* Hct-29.7* MCV-77* MCH-24.9*. Consequently, to work effectively with Bard, we preprocessed the text by removing special characters and retaining only alphanumeric characters.

### Prompting Strategies

Direct (standard prompting) and iterative prompting (chain of thought prompting) [27] are the 2 major prompting methods. Iterative prompting is a promising method for improving LLM performance on specialized tasks; however, it requires a predefined set of manually annotated reasoning steps, which can be time consuming and difficult to create, especially for specialized domains. Most users opt for a direct prompt method to save time and obtain an immediate response. Therefore, to analyze the effect of prompt formats on LLM performance, we assessed each model’s performance for every case using the 3 distinct direct prompt strategies outlined in Table 1. These strategies varied from open-ended to multiple-choice formats.

**Table 1:** Prompt strategies.

Approach	Prompt strategy description	Prompt sample
Approach 1 (open-ended prompt)	In this approach, prompts were formatted in an open-ended fashion. Formatting a prompt using this method allows the model to formulate a hypothesis for the case and explain why and what it thinks is the diagnosis. Here, we scored a model based on its ability to provide the correct diagnosis without additional assistance.	“What is the diagnosis? The case is: A 32-year-old man was evaluated in the emergency department of this hospital for the abrupt onset of postprandial chest pain...”
Approach 2 (multiple-choice prompt)	We formatted prompts as multiple-choice questions, and the LLMs^a^ were expected to select a single diagnosis from a list of options. The models were assigned a positive score in this task if they selected the correct diagnosis from the options.	“Choose the most likely diagnosis from the following: Option I: Cholecystitis, Option II: Acute coronary syndrome, Option III: Pericarditis, Option IV: Budd-Chiari syndrome. The case is: A 32-year-old man was evaluated in the emergency department of this hospital for the abrupt onset of postprandial chest pain...”
Approach 3 (ranking prompt)	The prompts were presented as a case and a list of diagnoses to be ranked by the LLMs. Models were assigned a positive score if the correct diagnosis was ranked first in this format.	“Rank the following diagnoses according to the most likely. Option I: Cholecystitis, Option II: Acute coronary syndrome, Option III: Pericarditis, Option IV: Budd-Chiari syndrome. The case is: A 32-year-old man was evaluated in the emergency department of this hospital for the abrupt onset of postprandial chest pain...”

^a^LLM: large language model.

Building upon prior research by Wang et al [28] and Li et al [29], we hypothesized that using a diverse range of prompts can reveal distinct reasoning paths while maintaining consistency in the correct responses regardless of the variations. When using multiple-choice prompts for the DC3 cases, we presented the same options available in the original web-based polls to the models, but on the MIMIC-III data set, we generated random wrong answers that were closely related to the correct diagnosis. We evaluated each LLM by assigning a positive or negative score (binary score) based on their responses. A positive score was assigned only if the models correctly selected the diagnosis for either data set. Conversely, we omitted the options for open-ended prompts, expecting the models to generate the correct diagnosis independently. Positive scores were awarded only if the models accurately provided the correct diagnosis.

### Prompt Ensemble: Majority Voting

**Figure 1:**
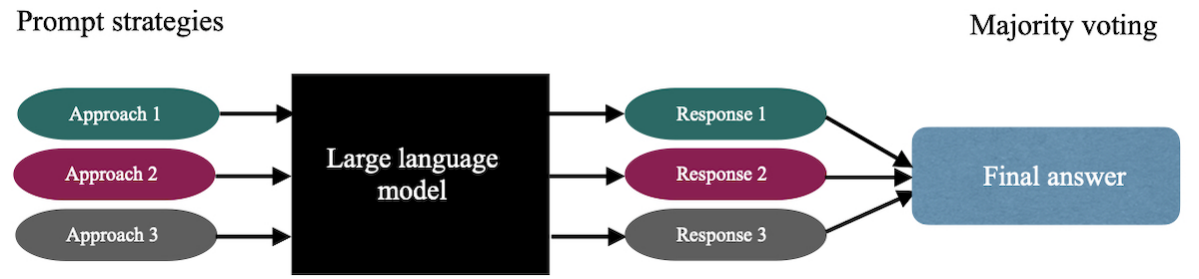
Our proposed method contains the following steps: (1) prompt a language model using a distinct set of prompts, (2) obtain diverse responses, and (3) choose the most consistent response as the final answer (majority voting).

To safely use imperfect language models, users must determine when to trust their predictions, particularly in critical situations, such as clinical decision support. Therefore, we used a majority voting (prompt ensembling) strategy to enhance the reliability of LLMs’ responses. The majority voting approach involves aggregating multiple responses and selecting the most common answer. By applying this approach to responses generated by different LLMs, we can observe the level of agreement and infer the consistency in their outputs for a given prompt. Specifically, we hypothesized that using a majority voting approach from the ensemble of prompt responses would boost the reliability of language models, minimizing potential errors, variations, and biases associated with individual prompting approaches. To achieve this, in independent chats, we prompted the LLM with 3 distinct prompt formats per case, as presented in Table 1. Subsequently, we collected the responses of each model and applied majority voting to aggregate its predictions, as presented in Figure 1. In majority voting, each prompt produced a response from the language model, and the majority response was chosen as the final response. In a scenario where all prompt strategies resulted in different responses, we assumed that the model was unsure of that question and scored the final response as a failure case. We limited the number of prompts in the ensemble to 3 because studies by Wang et al [28] and Li et al [29] have shown that we obtain diminishing returns as we increase the overall number of prompts in an ensemble.

### Ethical Considerations

No ethics approval was pursued for this research, given that the data was publicly accessible and deidentified. This aligns with the guidelines outlined in the National Institutes of Health investigator manual for human subjects research [30].

## Results

### Performance Across Prompt Strategies

**Figure 2:**
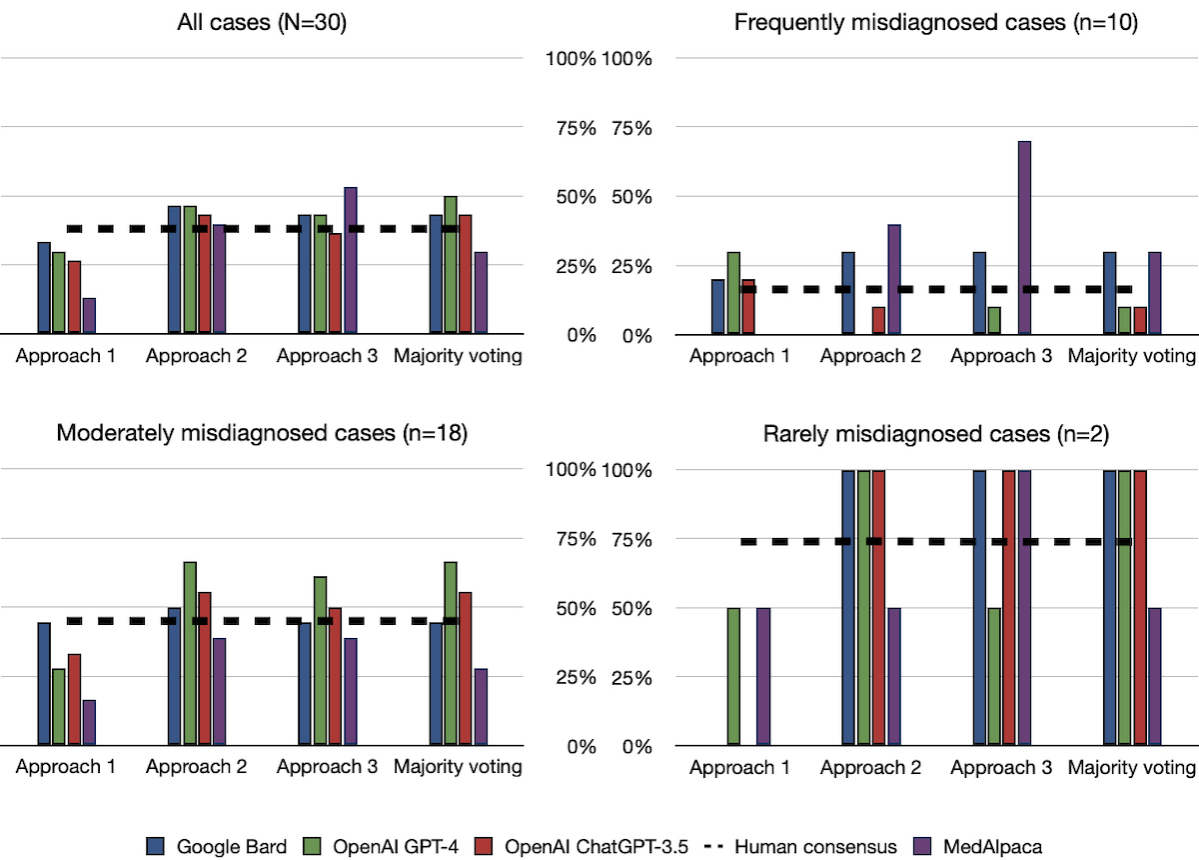
Results of the diagnostic case challenge collection data set comparing prompt strategies. OpenAI GPT-4 outperformed all other models, achieving the highest score in all 30 cases using the majority voting approach. Furthermore, all large language models except MedAlpaca outperformed the human consensus (denoted by a black dashed line) across all cases, regardless of the difficulty, using at least 1 prompt approach. GPT-4: generative pretrained transformer-4.

Figure 2 reveals the performance of LLMs across different prompts on the DC3 data set. Overall, approach 2 (multiple-choice prompt) yielded the highest score for all 30 cases, with GPT-4 and Bard achieving an accuracy score of 47% (14/30) and ChatGPT-3.5 obtaining a score of 43% (13/30). However, when considering case difficulty, the results varied. On the frequently misdiagnosed cases category, GPT-4 and ChatGPT-3.5 performed better with open-ended prompts (approach 1), scoring 30% (3/10) and 20% (2/10), respectively. In contrast, Bard demonstrated superior performance with multiple-choice prompts for selection and ranking (approaches 2 and 3), achieving a score of 30% (3/10). ChatGPT-3.5 and Bard performed equally well on the rarely misdiagnosed cases category using approaches 2 and 3, achieving a perfect score of 100% (2/2). Furthermore, GPT-4 attained a score of 100% (2/2) but only with approach 2. For the moderately misdiagnosed cases category, all LLMs achieved their best performance with approach 2, scoring 67% (12/18), 56% (10/18), and 50% (9/18) for GPT-4, ChatGPT-3.5, and Bard, respectively. Table S1 in the Multimedia Appendix 1 presents the inconsistencies in the correct responses across the approaches for different cases. For example, Bard could only diagnose milk alkali syndrome using approach 1 but failed to use other prompt approaches. ChatGPT-3.5 correctly diagnosed primary adrenal insufficiency (Addison disease) with only approach 2, whereas GPT-4 was able to diagnose acute hepatitis E virus infection with only approach 1. These results indicate that no universal prompt approach is optimal for all LLMs when dealing with complex cases.

**Figure 3:**
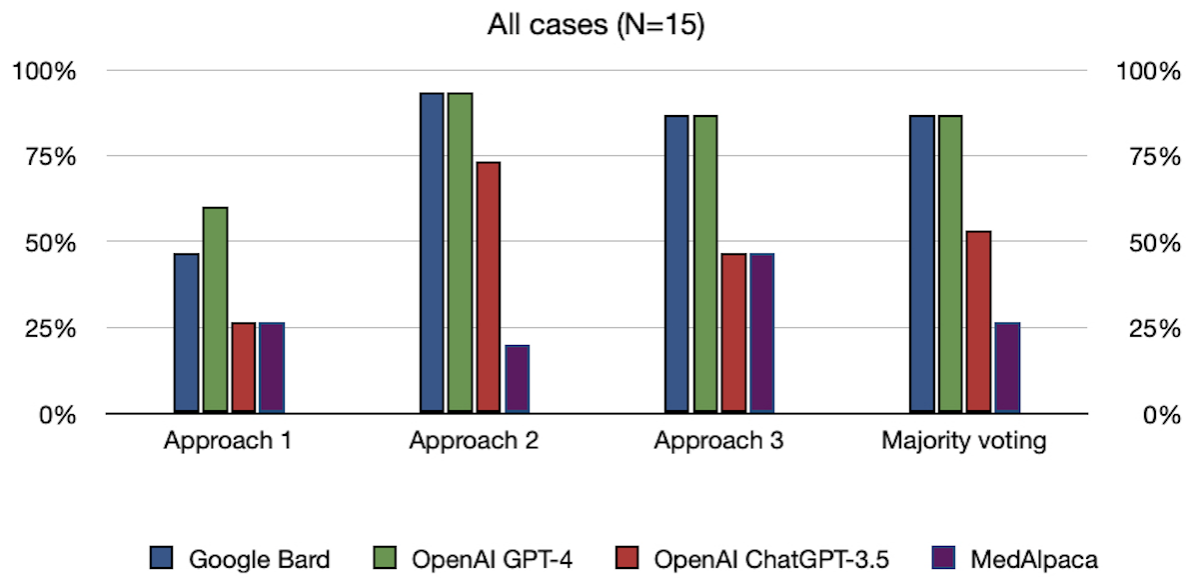
Results of the Medical Information Mart for Intensive Care-III data set across prompt strategies. Approach 1 (open-ended prompt) proved challenging for all the large language models compared with approach 2 (multiple-choice prompt) and approach 3 (ranking prompt).

Results on the MIMIC-III data set in Figure 3 showed that the LLMs also performed best using approach 2 (multiple-choice prompt), with Bard and GPT-4 obtaining scores of 93% (14/15) each and ChatGPT-3.5 obtaining 73% (11/15). Using approach 3 (ranking prompt) resulted in a slight drop in performance for GPT-4 and Bard, with a 6% decrease, whereas the performance of ChatGPT-3.5 dropped by 26%. Approach 1 (open-ended prompt) proved challenging for the LLMs, with scores of 47% (7/15), 60% (9/15), and 27% (4/15) for Bard, GPT-4, and ChatGPT-3.5, respectively. Table S2 in the Multimedia Appendix 1 illustrates that approach 1 was only beneficial to GPT-4 in diagnosing amyloidosis, whereas it was consistently never the sole correct approach for Bard and ChatGPT-3.5. These results aligned with the findings from the DC3 data set and emphasized the varying performances of different models and prompt approaches across tasks.

### Performance With Majority Voting

Previous experiments have demonstrated that there is no perfect prompting strategy because LLM users may not know beforehand which prompt will produce a correct response. We used the majority voting approach to estimate consistency, maximize the benefits of different prompt strategies, and enhance the reliability of the LLMs’ responses. Figure 2 illustrates the results for all DC3 cases. Majority voting improved the overall performance of GPT-4 from 47% to 50%, whereas the performance of ChatGPT-3.5 remained at 43% because majority voting did not decrease its performance compared with that of approach 2. In contrast, the performance of Bard decreased from 47% to 43% compared with that of approach 2. Summarizing the overall performance based on query difficulty, majority voting resulted in a perfect score of 100% for the rarely misdiagnosed cases category across all the LLMs. For the frequently misdiagnosed cases category in DC3, Bard achieved the highest score with majority voting and multiple-choice prompts, whereas GPT-4 performed best for the moderately misdiagnosed cases category with majority voting and approach 2. In addition, GPT-4 outperformed all other LLMs across all DC3 cases using the majority voting approach, regardless of the case difficulty. This score surpassed the performance of the individual prompt approaches in all cases.

Results on the MIMIC-III data set in Figure 3 showed that, the scores with majority voting were 87% (13/15) for GPT-4 and Bard each and 53% (8/15) for ChatGPT-3.5. These results indicate that the ensemble method did not substantially improve their performance compared with their best individual approach. It is worth noting that although the majority voting approach did not consistently outperform individual approaches in terms of the highest number of correct responses, it did provide a means to consolidate predictions and mitigate potential errors and biases from single approaches.

### Comparison With Human Respondents

In the DC3 cases, although the human respondents had the advantage of accessing supporting patient information such as image scans and magnetic resonance imaging, the LLMs consistently outperformed the average human consensus. As shown in Figure 2, using the majority voting approach, all LLMs achieved a higher performance than the human consensus (denoted by a black dashed line), with a minimum margin of 5% across all 30 cases. Specifically, when considering query difficulty, the LLMs demonstrated even greater superiority. In the rarely misdiagnosed cases category, all LLMs surpassed the average human consensus by a substantial margin of 26%. For the moderately misdiagnosed cases category, GPT-4 and ChatGPT-3.5 maintained their advantage over human respondents, achieving a minimum margin of 11% with the majority voting approach. In contrast, only Bard outperformed the human average consensus on the frequently misdiagnosed cases category, with a margin of 14%.

We conducted a Spearman rank correlation test to analyze the pattern in the responses between each LLM and the human respondents. This involved correlating the average percentage of correct responses for each LLM across the prompt strategies with that of correct human responses. The results of the Spearman correlation test revealed that Bard had a relatively weak correlation coefficient of 0.30, whereas GPT-4 and ChatGPT-3.5 exhibited moderate positive correlations of 0.51 and 0.50, respectively. This suggested that the diagnostic performance patterns of GPT-4 and ChatGPT-3.5 aligned moderately with those of the human respondents. The observed correlation in answering patterns between human respondents and LLMs may stem from the inherent data bias present in the training data sets. The LLMs learn from vast amounts of data, and if the training data are biased toward certain diagnostic or decision-making patterns commonly expressed by human physicians, the model is likely to replicate those patterns. Although the correlation suggested that the LLMs have the potential to be valuable tools in medical education, it is important to note their correlation with human physicians and that the performance of LLMs does not necessarily mean that they are as good as human physicians in diagnosing and treating diseases.

We could not directly compare the performance of human respondents on the MIMIC-III data sets because of the unavailability of data. Overall, the results indicated that the LLMs consistently outperformed the average human consensus in diagnosing medical cases, showcasing their potential as a tool to complement and enhance care quality and education for complex diagnostic cases.

### Comparison With MedAlpaca

On the DC3 data sets, Bard, GPT-4, and ChatGPT-3.5 outperformed MedAlpaca across all cases using the majority voting approach by a minimum margin of 13%. MedAlpaca also displayed the worst performance in the open-ended prompts, irrespective of query difficulty. However, when multiple-choice options were provided, MedAlpaca outperformed the other LLMs in the frequently misdiagnosed cases category. Similar to the DC3 data set, MedAlpaca consistently demonstrated its best performance using the ranking prompt on the MIMIC-III data sets. However, its overall performance was significantly poorer than the other LLMs, with each LLM outperforming the model by at least 26% using the majority voting approach. In contrast to the general-purpose LLMs (eg, Bard, GPT-4, and ChatGPT-3.5), investigating the MedAlpaca model was finetuned using diverse medical tasks and assessed using multiple-choice medical examinations. This tailored training approach likely contributed to its notable performance, particularly excelling in DC3 cases (frequently misdiagnosed instances) and demonstrating optimal results in multiple-choice queries.

### Qualitative Analysis

In our experiments, we manually observed the responses of each LLM to all our prompts and noted that each LLM consistently justified its diagnosis choice except for MedAlpaca. Specifically, each LLM offered a logical explanation for its chosen response regardless of the prompting strategy. For further investigation, we analyzed each LLM’s responses in 3 scenarios: (1) when presented with multiple-choice options containing the true diagnosis and they responded accurately, (2) when their response was incorrect, and (3) when given only incorrect multiple-choice options to pick from. In the first scenario, as presented in Multimedia Appendix 1, all LLMs (eg, Bard, GPT-4, and ChatGPT-3.5) mentioned that their rationale for diagnosing *miliary tuberculosis* was owing to relevant symptoms presented in the case, such as a *history of respiratory illness and the presence of mesenteric lymph nodes and numerous tiny nodules throughout both lungs distributed in a miliary pattern.* This pattern of offering insightful reasons for the likelihood of a diagnosis and explaining why other diagnostic options are less probable is valuable for educational purposes. In the second scenario, we observed that there was a notable disparity in the accuracy of human respondents. Only 6% (217/3624) of the human participants provided the correct response, with most votes (1232/3624, 34%) favoring *ulcerative colitis*, whereas 23% (833/3624) of the human responses opted for *salmonellosis*. Notably, Bard and GPT-4 displayed similar behavior by selecting salmonellosis, whereas ChatGPT-3.5 and MedAlpaca chose *ulcerative colitis*.

Another notable finding occurred in the responses of GPT-4 and ChatGPT-3.5. Regardless of the correctness of their chosen diagnoses, these models consistently recommended further tests to confirm their responses. This behavior suggested a general tendency toward advocating additional examinations to validate their diagnoses, potentially reflecting a cautious approach. In contrast, Bard adopted a different approach. Instead of recommending further tests, Bard highlighted that the provided query information supported the diagnosis without suggesting additional confirmatory measures. In the scenario where only incorrect options were given, Bard, ChatGPT-3.5, and MedAlpaca made choices and justified their responses. In contrast, GPT-4 explicitly mentioned that none of the provided options matched the case presentation. Furthermore, GPT-4 suggested a more probable diagnosis and recommended additional testing to explore its feasibility.

## Discussion

### Principal Findings

Previous studies [4,5] have presented the impressive success of LLMs in standardized medical examinations. We conducted experiments to assess the potential of LLMs as a CME system for rare and complex diagnoses, and our findings demonstrated that LLMs have the potential to be a valuable tool for rare disease education and differential diagnosis. Although LLMs demonstrated superior performance compared with the average human consensus in diagnosing complex diseases, it is essential to note that this does not imply their superiority over physicians. Numerous unknown factors, including the level of respondents’ expertise, may influence the outcome of web-based polls. Furthermore, we examined the knowledge capacity of LLMs through open-ended and multiple-choice prompts and found that LLMs, including MedAlpaca, performed better with multiple-choice prompts. This improvement can be attributed to the options provided, which narrowed the search space for potential diagnoses from thousands to a few likely possibilities. Consequently, we surmise that LLMs are not yet ready to be used as stand-alone tools, which aligns with the findings of previous studies [5,17,18]. Our observations revealed the consistent outperformance of general-purpose LLMs over MedAlpaca in various experiments. Their superior ability to provide valuable justifications for making diagnoses was particularly noteworthy, a strength not matched by MedAlpaca. This difference may stem from MedAlpaca’s exclusive finetuning and assessment for multiple-choice medical examinations, which slightly differ in format from the clinical cases in our experiments.

A notable finding in the response of LLMs to queries was their consistent provision of coherent and reasoned explanations, regardless of the query format. For instance, when diagnosing *miliary tuberculosis*, all 3 LLMs emphasized that the patient’s systemic symptoms, exposure risks, chest radiograph, computed tomography scan findings, and the suspected compromised immune state collectively support the diagnosis of *miliary tuberculosis.* Furthermore, Bard and GPT-4 ruled out other diagnoses presented in the multiple-choice prompt by highlighting their less typical presentations and lack of certain associated symptoms or risk factors. In addition, the conversational nature of LLMs allows users to ask follow-up questions for further context. These attributes hold great potential for educating users and offering them insights. However, we observed that LLMs provided logical explanations, even when their diagnoses were incorrect. ChatGPT-3.5 and GPT-4 may suggest additional testing to validate their selected diagnosis or use cautious terms like “potential diagnosis.” However, it remains unclear whether these recommendations stem from the models’ internal confidence or whether there are features intentionally designed by the developers for cautious use. The absence of explicit information regarding the level of uncertainty of LLMs for a specific case is concerning as it could potentially mislead clinicians. The ability to quantify uncertainty is crucial in medical decision-making, in which accurate diagnoses and treatment recommendations are paramount. Clinicians heavily rely on confidence levels and probability assessments to make informed judgments [29]. Without an indication of uncertainty, there is a risk that clinicians may trust the logical explanations provided by the LLMs even when they are incorrect, leading to misdiagnoses or inappropriate treatment plans.

Considering the delicate role of clinical decision support, it is essential to address validity and reliability as crucial aspects of uncertainty. Moreover, a reliable system is of paramount importance for medical education. However, the stochastic nature of LLMs introduces doubts among clinicians regarding their reliability. Although a specific metric to quantitatively assess the reliability of the LLMs used in this study is currently lacking, we acknowledge the significance of consistency in achieving reliability. To address this, we used different prompting strategies and implemented a majority voting approach to select the most consistent response from each LLM. After examining the individual prompt strategies, we anticipated consistent responses across strategies for a specific case. However, our findings revealed that the responses of LLMs were sensitive to concrete prompt formats, particularly in complex diagnoses. For instance, ChatGPT-3.5 and GPT-4 performed better with the open-ended prompt (approach 1) in the frequently misdiagnosed cases category of DC3 cases but struggled with similar cases using multiple-choice and ranking prompts (approaches 2 and 3). In contrast, Bard performed better with multiple-choice prompts. These results highlighted that there is no one-size-fits-all prompting approach nor does a single strategy apply universally to all LLMs. Although the majority voting strategy did not yield optimal results for all models across data sets, it served as a means to consolidate responses from multiple prompts and provided a starting point for incorporating reliability.

Several studies [10-12,14,15] have emphasized the significance of enhancing the education of clinicians at all levels to provide better support for rare and complex diagnoses. In this pursuit, the studies by Lee et al [8] and Decherchi et al [31] have highlighted the potential advantages of artificial intelligence (AI) systems, whereas the studies by Abdullahi et al [25] and Sutton et al [32] have reported a lack of acceptance of AI tools among clinicians. For instance, younger medical students and residents appeared more receptive to integrating technology [33]. One notable reason for this lack of acceptance is that conventional AI systems typically require training before clinicians can effectively use them, which can be burdensome and time consuming [32]. In contrast, conversational LLMs, such as ChatGPT-3.5, Bard, and GPT-4, offer a distinct advantage with their simple interface and dialogue-based nature. These conversational LLMs eliminate the need for extensive training, increasing their potential for high acceptance across all levels of medical practice. Although the exciting ease of use, conversational nature, impressive display of knowledge, and logical explanations of LLMs have the potential for user education and insights, their current limitations in reliability and expressing uncertainty must be addressed to ensure their effective and responsible use in critical domains, such as health care.

### Limitations

First, the limitations of the knowledge of ChatGPT-3.5 and GPT-4 to the latest trends and updates in health care (or medical) data till 2021 pose the risk of potentially incomplete information and hamper the effectiveness of the models as a CME tool, especially when addressing emerging diseases. In contrast, although continuous updates to Bard are advantageous for keeping the model up-to-date, this attribute may impact the reproducibility of our study. Second, it is notable that our experiments had a limited scope owing to a small sample size consisting of only 30 diseases from the DC3 data set and 15 cases from the MIMIC-III data set. In addition, although we took precautions to preprocess the MIMIC-III notes to prevent leakage of the final diagnosis, the discharge summaries may still contain nuanced information that could make the diagnosis obvious. Furthermore, the closed nature of the LLMs used in this study restricted our technique for measuring reliability to a majority voting approach, which consolidated responses from diverse prompts. Although majority voting can help to mitigate the variability of LLM output, it is notable that LLMs may still generate different responses for the same prompt. This variability should be considered when interpreting the results of this study. However, when these LLMs are released with an enhanced iteration that allows for finetuning and calibration, future work should incorporate more effective mechanisms to estimate and communicate uncertainty. An example of such an approach could involve assigning a confidence score to the probability score of their responses. This methodology could allow clinicians to make informed decisions regarding whether to accept or reject responses that fall within a desired threshold.

### Conclusions

In this study, we conducted experiments to assess the potential of LLMs, including ChatGPT-3.5, GPT-4, and Bard, as a CME system for rare and complex diagnoses. First, we evaluated their diagnostic capability specifically for rare and complex cases. Subsequently, we explored the impact of prompt formatting on their performance. Our results revealed that these LLMs possessed potential diagnostic capacities for rare and complex medical cases, surpassing the average crowd consensus on the DC3 cases. For selected rare cases from the MIMIC-III data set, Bard and GPT-4 achieved a diagnostic accuracy of 93%, whereas ChatGPT-3.5 achieved an accuracy of 73%. Our findings highlighted that users might discover an approach that yields favorable results for various queries by exploring different prompt formats. In contrast, using majority voting of responses from multiple prompt strategies offers the benefit of a robust and reliable model, instilling confidence in the generated responses. However, determining the best prompt strategy versus relying on the majority voting approach involves a tradeoff between exploration and exploitation. Although prompt engineering research is continuing, we hope that future studies will yield better solutions to enhance the reliability and consistency of the responses of LLMs. Overall, our study’s results and conclusions provide a benchmark for the performance of LLMs and shed light on their strengths and limitations in generating responses, expressing uncertainty, and providing diagnostic recommendations. The insights gained from this study can serve as a foundation for further exploration and research on using LLMs as medical education tools to enhance their performance and capabilities as conversational language models.

## Appendix

Multimedia Appendix 1 Comprehensive tables detailing the performance of each model across data sets, with included examples of prompts and responses for each model.

## Abbreviations

AI: artificial intelligence
CME: continuing medical education
DC3: diagnostic case challenge collection
LLM: large language model
MIMIC-III: Medical Information Mart for Intensive Care-III
USMLE: United States Medical Licensing Examination

## Data Availability

The URLs for the diagnostic case challenge collection data set can be obtained via A Diagnostic Case Challenge Collection [34]. The Medical Information Mart for Intensive Care data sets can be accessed via the database, Medical Information Mart for Intensive Care-III Clinical Database v1.4 [35], after obtaining permission from Physionet.
