# Supplementary material for: Learning to Make Rare and Complex Diagnoses With Generative AI Assistance: Qualitative Study of Popular Large Language Models
Source: JMIR Med Educ. 2024 Feb 13;10:e51391. doi: 10.2196/51391 (PMC10900078; doi:10.2196/51391)
Supplement: Multimedia Appendix 1 [file mededu_v10i1e51391_app1.docx]

**Multimedia Appendix 1**

**Table S1.** Detailed view of the results obtained by Bard (in blue), GPT-4^a^ (in green), ChatGPT-3.5 (in red), and MedAlpaca (in black) across cases for the open-ended task (approach 1) and multiple-ended tasks (approach 2 and task 3) from the DC3^b^ data set.

| **S/No** | **Diagnosis** | **Case category** | **LLMs^c^ responses** | | | | **Human responses** |
| --- | --- | --- | --- | --- | --- | --- | --- |
|  |  |  | **Approach 1** | **Approach 2** | **Approach 3** | **Majority voting across approaches** | **Correct human respondents.**  **(n/N%)** |
| **1** | **Insulinoma** | **Rarely misdiagnosed** | **XXXX** | **✔✔✔X** | **✔✔✔✔** | **✔✔✔X** | **76.00**  **(8344/10,979)** |
| **2** | **Miliary tuberculosis** | **Rarely misdiagnosed** | **X✔X✔** | **✔✔✔✔** | **✔X✔✔** | **✔✔✔✔** | **70.00**  **(4526/6465)** |
| **3** | **Neisseria meningitidis infection and terminal complement deficiency** | **Moderately misdiagnosed** | **✔X✔✔** | **✔✔X✔** | **✔✔✔X** | **✔✔✔✔** | **56.99**  **(1643/2883)** |
| **4** | **Lemierre's syndrome** | **Moderately misdiagnosed** | **X✔XX** | **✔✔✔X** | **✔✔✔X** | **✔✔✔X** | **56.99**  **(4377/7679)** |
| **5** | **Leakage of a hydatid cyst.** | **Moderately misdiagnosed** | **XXXX** | **X✔✔X** | **X✔✔X** | **X✔✔X** | **57.00**  **(5404/9480)** |
| **6** | **Lead poisoning** | **Moderately misdiagnosed** | **✔XXX** | **X✔X✔** | **X✔X✔** | **X✔XX** | **53.99**  **(1860/3445)** |
| **7** | **Secondary syphilis** | **Moderately misdiagnosed** | **XX✔X** | **XX✔X** | **✔X✔✔** | **XX✔X** | **52.00**  **(1208/2323)** |
| **8** | **Digoxin toxicity** | **Moderately misdiagnosed** | **✔XXX** | **✔✔✔✔** | **X✔✔✔** | **✔✔✔✔** | **51.00**  **(5409/10605)** |
| **9** | **Intestinal tuberculosis** | **Moderately misdiagnosed** | **XXXX** | **XXXX** | **XXXX** | **XXXX** | **49.01**  **(1069/2181)** |
| **10** | **Disseminated mycobacterium bovis infection** | **Moderately misdiagnosed** | **X✔XX** | **X✔XX** | **✔✔XX** | **X✔XX** | **46.00**  **(1980/4304)** |
| **11** | **Perforation of the right ventricular wall by an implantable cardioverter–defibrillator lead** | **Moderately misdiagnosed** | **XXXX** | **XXX✔** | **XXXX** | **XXXX** | **45.00**  **(2376/5280)** |
| **12** | **Primary neurosarcoidosis** | **Moderately misdiagnosed** | **✔✔✔X** | **✔✔✔X** | **✔✔✔X** | **✔✔✔X** | **42.98**  **(732/1703)** |
| **13** | **Necrotizing lymphadenitis (Kikuchi–Fujimoto disease)** | **Moderately misdiagnosed** | **✔XXX** | **✔✔✔✔** | **✔✔✔✔** | **✔✔✔✔** | **42.00**  **(2095/4987)** |
| **14** | **Congenital rubella syndrome** | **Moderately misdiagnosed** | **✔✔✔X** | **✔✔✔X** | **XXXX** | **✔✔✔X** | **42.01**  **(2767/6587)** |
| **15** | **Thrombotic thrombocytopenic purpura** | **Moderately misdiagnosed** | **XXX✔** | **XXXX** | **XXX✔** | **XXX✔** | **40.00 (2988/7470)** |
| **16** | **Primary adrenal insufficiency (Addison’s disease)** | **Moderately misdiagnosed** | **XXXX** | **✔X✔X** | **XXX✔** | **XXXX** | **37.00**  **(2464/6659)** |
| **17** | **Primary testicular lymphoma** | **Moderately misdiagnosed** | **X✔✔X** | **X✔✔X** | **X✔✔✔** | **X✔✔X** | **37.00 (2357/6371)** |
| **18** | **Abdominal migraine** | **Moderately misdiagnosed** | **✔X✔✔** | **✔✔X✔** | **✔✔XX** | **✔✔X✔** | **36.00**  **(1619/4496)** |
| **19** | **IgA vasculitis** | **Moderately misdiagnosed** | **✔XXX** | **✔✔✔✔** | **✔✔✔X** | **✔✔✔X** | **33.98**  **(1906/5606)** |
| **20** | **Measles** | **Moderately misdiagnosed** | **XXXX** | **XXXX** | **XXX✔** | **XXXX** | **32.00**  **(2104/6576)** |
| **21** | **Androgen insensitivity syndrome** | **Frequently misdiagnosed** | **XXXX** | **XXXX** | **XXXX** | **XXXX** | **27.00**  **(1755/6499)** |
| **22** | **Leptospirosis, acute leptospirosis** | **Frequently misdiagnosed** | **✔✔XX** | **✔XXX** | **✔XXX** | **✔XXX** | **24.00**  **(1635/6811)** |
| **23** | **Acute cholecystitis or choledocholithiasis** | **Frequently misdiagnosed** | **XXXX** | **XXXX** | **XXX✔** | **XXXX** | **22.00**  **(2433/11059)** |
| **24** | **Acute HEV infection** | **Frequently misdiagnosed** | **X✔✔X** | **✔X✔✔** | **✔XX✔** | **✔X ✔✔** | **20.00**  **(706/3529)** |
| **25** | **Disseminated pulmonary blastomycosis** | **Frequently misdiagnosed** | **XXXX** | **✔XX✔** | **✔XX✔** | **✔XX✔** | **18.00**  **(1121/6227)** |
| **26** | **Milk alkali syndrome** | **Frequently misdiagnosed** | **✔XXX** | **XXX✔** | **XXXX** | **XXXX** | **17.00**  **(701/4124)** |
| **27** | **Borrelia miyamotoi infection** | **Frequently misdiagnosed** | **X✔✔X** | **XXXX** | **X✔X✔** | **X✔XX** | **13.00**  **(586/4505)** |
| **28** | **Wilson’s disease** | **Frequently misdiagnosed** | **XXXX** | **XXX✔** | **XXX✔** | **XXX✔** | **8.00**  **(377/4715)** |
| **29** | **Hodgkin’s lymphoma** | **Frequently misdiagnosed** | **XXXX** | **XXXX** | **XXX✔** | **XXXX** | **5.99 (217/3624)** |
| **30** | **Infective endocarditis** | **Frequently misdiagnosed** | **XXXX** | **XXXX** | **XXX✔** | **XXXX** | **2.04**  **(166/8132)** |

^a^GPT-4: generative pretrained transformer-4.

^b^DC3: diagnostic case challenge collection.

^c^LLM: large language model.

**Table S2.** Detailed view of the results obtained by Bard (in blue), GPT-4^a^ (in green), ChatGPT-3.5 (in red), and MedAlpaca (in black) across cases for the open-ended task (approach 1) and multiple-ended tasks (approach 2 and task 3) for the MIMIC-III^b^ data sets.

| **S/No** | **Diagnosis** | **LLMs^c^ responses** | | | |
| --- | --- | --- | --- | --- | --- |
|  |  | **Approach 1** | **Approach 2** | **Approach 3** | **Majority voting across Approaches** |
| 1 | Stevens-Johnson Syndrome | ✔✔**XX** | ✔✔✔✔ | ✔✔✔✔ | ✔✔✔✔ |
| 2 | Adenocarcinoma of the ampulla of vater. | ✔**XX**✔ | ✔✔**XX** | ✔**XX**✔ | ✔**XX**✔ |
| 3 | Babesiosis | ✔**XXX** | ✔✔✔**X** | ✔✔**XX** | ✔✔**XX** |
| 4 | Pituitary tumor | ✔✔**X**✔ | ✔✔✔✔ | ✔✔✔✔ | ✔✔✔✔ |
| 5 | Methemoglobinemia | ✔✔✔✔ | ✔✔✔✔ | ✔✔✔✔ | ✔✔✔✔ |
| 6 | Myasthenia gravis crisis | **X**✔✔✔ | ✔✔✔**X** | ✔✔✔**X** | ✔✔✔**X** |
| 7 | Myelodysplastic syndrome | **XXXX** | ✔✔**XX** | **X**✔**XX** | **X**✔**XX** |
| 8 | Guillain-Barré syndrome | **X**✔**XX** | ✔✔✔**X** | ✔✔**XX** | ✔✔**XX** |
| 9 | Amyloidosis | **X**✔**XX** | ✔**XXX** | ✔**XX**✔ | ✔**XXX** |
| 10 | Thrombocytopenia | ✔✔**XX** | ✔✔✔**X** | ✔✔✔✔ | ✔✔✔**X** |
| 11 | Intrahepatic cholangiocarcinoma | **XXXX** | ✔✔**XX** | ✔✔**XX** | ✔✔**XX** |
| 12 | Cushing syndrome | ✔✔✔**X** | ✔✔✔**X** | ✔✔✔**X** | ✔✔✔**X** |
| 13 | Heparin-Induced thrombocytopenia | **X**✔✔**X** | ✔✔✔**X** | ✔✔**XX** | ✔✔✔**X** |
| 14 | Hodgkin’s lymphoma | **XXXX** | **X**✔✔**X** | **X**✔**X**✔ | **X**✔**X X** |
| 15 | Non-Hodgkin’s lymphoma | **XXXX** | ✔✔✔**X** | ✔✔✔**X** | ✔✔✔**X** |

^a^GPT-4: generative pretrained transformer-4

^b^MIMIC-III: Medical Information Mart for Intensive Care-III.

^c^LLM: large language model.

**Qualitative Analysis**

We qualitatively analyzed the LLMs’ responses in three scenarios: (i) when presented with multiple-choice options containing the true diagnosis, and they responded accurately; (ii) when their response was incorrect; (iii) when given only incorrect multiple-choice options. The highlighted text in orange is the input prompt, while the text highlighted in red, green, blue, and black are ChatGPT-3.5, GPT4, Bard, and MedAlpaca’s responses, respectively.

**Example Prompt:**

Choose the most likely diagnosis from the following and explain the rationale for your answer: Option I: Visceral Kaposi's sarcoma, Option II: Gastric lymphoma with hemorrhage, Option III: Disseminated histoplasmosis, Option IV: Miliary tuberculosis. The case is : A 29 year old man was seen in an outpatient clinic affiliated with this hospital because of abdominal pain fever and weight loss The patient was reportedly well until unintentional weight loss occurred approximately 6 weeks before presentation Three weeks before presentation upper abdominal pain developed that the patient rated at 8 on a scale of 0 to 10 with 10 indicating the most severe pain The pain radiated to his throat increased after eating and drinking and did not diminish with ibuprofen At the outpatient clinic the patient reported nausea occasional vomiting decreased food intake and a loss of 10 kg from his usual weight of 4 kg He reported no headaches visual changes neck stiffness dyspnea substernal chest pain lower abdominal pain diarrhea back pain dysuria rashes or joint pain He had had a respiratory illness approximately 2 years before this evaluation He had no known allergies He was born in Central America and had been living in the United States for 4 years He worked in agriculture and did not speak English He smoked cigars occasionally drank alcohol infrequently and did not use illicit drugs On examination the patient appeared cachectic The temperature was 3 C the blood pressure 101 68 mm Hg the pulse 114 beats per minute the respiratory rate 20 breaths per minute and the oxygen saturation 100 while he was breathing ambient air The abdomen was soft with normal bowel sounds and without tenderness or distention There was macular hyperpigmentation of the feet and legs that reportedly had not changed for 10 years The remainder of the examination was normal Urinalysis revealed 1 protein and was otherwise normal Ranitidine was prescribed and the patient returned home with instructions to follow up the next day when test results were available Blood levels of glucose calcium phosphorus lipase and amylase were normal as were the results of tests of liver and renal function Testing for antibodies to hepatitis C virus was negative other test results are shown in A frontal chest radiograph showed subtle tiny nodules throughout both lungs without focal consolidation 1A There was no evidence of mediastinal or hilar lymphadenopathy Two days later the patient returned to the outpatient clinic He reported persistent abdominal pain that he rated at 8 on a scale of 0 to 10 as well as sore throat fever and one episode of hematemesis that had occurred the day before He reported no cough congestion or improvement with ranitidine The temperature was 3 C the blood pressure 105 65 mm Hg the pulse 119 beats per minute the respiratory rate 18 breaths per minute and the oxygen saturation 99 while he was breathing ambient air There were coarse breath sounds at both lung bases The abdomen was firm without distention masses guarding or rebound the remainder of the examination was unchanged Airborne precautions were instituted and a face mask was placed Acetaminophen was administered and a tuberculin skin test was performed The patient was transported to the emergency department of this hospital He was heterosexual and reported having no more than eight partners in his lifetime and consistently using condoms There was no known history of exposure to tuberculosis On examination the temperature was 9 C the blood pressure 100 65 mm Hg the pulse 68 beats per minute the respiratory rate 24 breaths per minute and the oxygen saturation 99 while the patient was breathing ambient air The weight was 6 kg the height 160 cm and the body mass index the weight in kilograms divided by the square of the height in meters A white exudate was present on the underside of the tongue A nontender mobile lymph node 1 to 2 cm in diameter was present in the left submandibular chain and additional nontender mobile lymph nodes were present in the right submental region and the right cervical region Fine rales were scattered throughout both lungs and the abdomen was soft the remainder of the examination was unchanged Blood levels of glucose calcium phosphorus magnesium lipase and amylase were normal as were the results of tests of liver and renal function other test results are shown in Computed tomography CT of the chest performed after the intravenous administration of contrast material revealed numerous nodules 1 to 3 mm in diameter randomly distributed in a miliary pattern i e having an appearance that is similar to millet seeds in both lungs without mediastinal hilar or axillary lymphadenopathy 1B and 1C To help evaluate the patient s abdominal pain a contrast enhanced CT scan of the abdomen and pelvis was obtained that showed multiple enlarged mesenteric lymph nodes and new small bilateral pleural effusions The mesenteric lymph nodes had low attenuation centers that were suggestive of central necrosis 1D There was no evidence of bowel dilatation or bowel wall thickening Specimens of blood sputum and urine were obtained Intravenous saline and one dose of ceftriaxone and azithromycin were administered followed by fluconazole omeprazole and nystatin suspension Results of coagulation tests were normal The patient was admitted to the hospital During the first day the temperature rose to 7 C Acetaminophen was administered

**ChatGPT Response:**

Based on the provided information, the most likely diagnosis for this patient is Option IV: Miliary tuberculosis. Here's the rationale for this answer:

1. Clinical Presentation: The patient presented with a constellation of symptoms suggestive of a systemic infectious disease. He had unintentional weight loss, fever, abdominal pain, and gastrointestinal symptoms like nausea, vomiting, and hematemesis.

2. Travel History and Occupation: The patient was born in Central America and had been living in the United States for 4 years. He worked in agriculture. This information is relevant because tuberculosis is more prevalent in certain regions, including Central America, and occupational exposure can increase the risk of infection.

3. Respiratory Illness in the Past: The patient had a history of respiratory illness approximately 2 years before the evaluation. This history raises suspicion for a possible prior exposure to tuberculosis.

4. Physical Examination Findings: The patient appeared cachectic and had coarse breath sounds in both lung bases. These findings suggest lung involvement, which is consistent with miliary tuberculosis.

5. Chest Radiograph Findings: The frontal chest radiograph showed subtle tiny nodules throughout both lungs without focal consolidation. This pattern is characteristic of miliary tuberculosis, where the infection disseminates hematogenously and results in widespread tiny nodules in the lungs.

6. Computed Tomography (CT) Findings: The CT scan of the chest revealed numerous nodules in a miliary pattern without mediastinal, hilar, or axillary lymphadenopathy. Again, these findings are consistent with miliary tuberculosis.

7. Abdominal CT Findings: The contrast-enhanced CT scan of the abdomen showed multiple enlarged mesenteric lymph nodes and small bilateral pleural effusions. The low attenuation centers of the mesenteric lymph nodes suggestive of central necrosis are consistent with tuberculosis.

8. Immunocompromised Host: The patient's immunocompromised state is suggested by his cachexia, which may be due to the underlying tuberculosis infection. Immunocompromised individuals, including those with HIV infection or other conditions affecting the immune system, are at higher risk of developing disseminated tuberculosis.

9. Response to Acetaminophen: The patient's temperature rose despite acetaminophen administration. This lack of response to antipyretics is commonly seen in tuberculosis.

Given the patient's clinical presentation, imaging findings, and risk factors, miliary tuberculosis is the most likely diagnosis. However, further diagnostic tests, such as acid-fast bacilli staining, mycobacterial culture, and molecular tests (e.g., PCR), would be necessary to confirm the diagnosis definitively.

**GPT4’s Response:**

Based on the presented information, Option IV: Miliary Tuberculosis seems the most likely diagnosis. The key reasons are:

1. The patient's symptoms: Fever, weight loss, abdominal pain, and cough are all common symptoms of tuberculosis (TB). These systemic symptoms are characteristic of a disseminated infection such as miliary TB.

2. The patient's imaging findings: The report of the chest CT scan mentioned numerous tiny nodules throughout both lungs distributed in a miliary pattern, which is a classic radiographic finding in miliary TB.

3. The patient's social history: The patient was born in Central America and is working in agriculture, both of which are risk factors for TB. Central America has a higher prevalence of TB than the United States, and working in agriculture could indicate potential exposure to migrant workers, a population often at high risk for TB.

4. Epidemiology: TB is more common than the other listed conditions, which are generally rare, especially in a relatively young patient without any other specified risk factors.

While each of the conditions listed could cause weight loss, fever, and abdominal pain, the CT findings and the patient's epidemiologic risk factors make miliary tuberculosis the most likely diagnosis. The others are less likely:

- Option I: Visceral Kaposi's sarcoma is often seen in HIV positive individuals, typically presenting with skin lesions, which this patient does not have. Also, it doesn't typically cause a miliary pattern on CT.

- Option II: Gastric lymphoma with hemorrhage could cause weight loss, abdominal pain, and hematemesis, but would not explain the lung nodules.

- Option III: Disseminated histoplasmosis could potentially cause similar symptoms and miliary nodules on imaging, but it's less likely given that the patient's job in agriculture didn't specify exposure to bird or bat droppings, a common source of histoplasmosis. The patient's lack of respiratory symptoms also makes this less likely.

Note that while miliary TB is the most likely diagnosis, it would be necessary to confirm this diagnosis with further testing such as a sputum smear or culture, or PCR for Mycobacterium tuberculosis. Additionally, the patient should be tested for HIV, as this can predispose to disseminated forms of TB and other opportunistic infections. His history of a respiratory illness 2 years prior and current presentation could possibly suggest undiagnosed/untreated HIV infection.

**Bard’s Response:**

The most likely diagnosis is Miliary tuberculosis. The patient presents with a history of weight loss, fever, and abdominal pain. He also has a history of a respiratory illness, which is a common presentation for tuberculosis. The physical examination findings, including the presence of lymphadenopathy and miliary nodules on chest imaging, are also consistent with tuberculosis. The other possible diagnoses are less likely. Visceral Kaposi's sarcoma is a rare cancer that is associated with HIV/AIDS. The patient does not have HIV/AIDS, so this diagnosis is less likely. Gastric lymphoma with hemorrhage is a rare type of cancer that can cause abdominal pain and hematemesis. However, the patient does not have any other symptoms of lymphoma, such as lymphadenopathy or night sweats. Disseminated histoplasmosis is a fungal infection that can cause fever, weight loss, and lymphadenopathy. However, the patient's chest imaging findings are not consistent with disseminated histoplasmosis.The patient's tuberculin skin test was negative. However, a negative tuberculin skin test does not rule out tuberculosis, especially in patients with advanced disease. The patient was started on treatment for tuberculosis, and his symptoms improved. This further supports the diagnosis of tuberculosis.

**Example Prompt 2:**

Choose the likely diagnosis from the following and explain the rationale for your answer: Option I: Ulcerative colitis, Option II: Hodgkin's lymphoma, Option III: Polyarteritis nodosa, Option IV: Salmonellosis. The case is: A 12 year old boy was admitted to this hospital because of malaise fevers abdominal pain and worsening pallor The patient had been well until 1 month before admission when headache and abdominal pain developed Examination by a school nurse revealed a temperature of 6 C and pale skin A diagnosis of a viral illness was made and follow up with his pediatrician was recommended When the patient was reexamined by the nurse 4 days later the temperature was normal The skin was pale the pharynx was slightly erythematous and the abdomen was tender At follow up visits with the school nurse 10 and 6 days before admission the patient reported persistent upper abdominal pain the temperature was 6 C at both visits On the day of admission the patient reported headache nausea and pain in the right upper abdomen and left leg The temperature was 3 C and the skin appeared to be more pale He was taken by ambulance to the emergency department of another hospital where he reported nausea occasional diarrhea and abdominal pain On examination he appeared mildly ill The temperature was 8 C the blood pressure 92 58 mm Hg the pulse 123 beats per minute the respiratory rate 18 breaths per minute and the oxygen saturation 98 while he was breathing ambient air The height was 132 cm 1st percentile for his age the weight 8 kg 1st percentile the weight had decreased 1 kg in 1 month and the body mass index the weight in kilograms divided by the square of the height in meters 4 9th percentile The skin was pale warm and dry The abdomen was soft and nontender and there was no hepatosplenomegaly the remainder of the examination was normal Blood levels of glucose calcium alanine aminotransferase alkaline phosphatase and total and direct bilirubin were normal as were results of renal function tests other test results are shown in Normal saline acetaminophen and oxygen through a nasal cannula at a rate of 2 liters per minute were administered the tachycardia resolved The patient was transferred by ambulance to this hospital On evaluation in the emergency department the patient reported a mild cough myalgias fatigue that had been worsening for 1 month and occasional dizziness during the previous 2 weeks He also reported nearly constant periumbilical pain that occasionally radiated to the left lower quadrant he described the pain as squeezing and rated it at up to 7 on a scale of 0 to 10 with 10 indicating the most severe pain He had daily loose stools that were green with occasional blood mixed in and on the toilet paper but he had no increased bruising or bleeding when he brushed his teeth He had been previously healthy and his immunizations were reportedly current with the exception of a seasonal influenza vaccine He took no medications and had no known allergies He had been born in the Caribbean had come to the United States 4 years earlier was a student and lived with his parents and two siblings He had visited the Dominican Republic including a rural village 3 months before admission he had not taken malaria prophylaxis He had pet fish and no exposures to animals or sick contacts There was no family history of sickle cell disease other hereditary anemia inflammatory bowel disease or autoimmune diseases On examination the patient was thin and appeared younger than his age The vital signs and oxygen saturation while he was breathing ambient air were normal The conjunctiva and oral mucosa were pale There was no palpable peripheral lymphadenopathy The abdomen was distended with mild tenderness on palpation in the right upper and left lower quadrants there were ecchymoses on the shins and the remainder of the examination was normal A stool specimen showed no occult blood. Blood levels of glucose phosphorus magnesium calcium alkaline phosphatase alanine aminotransferase and total and direct bilirubin were normal as were results of renal function tests Other test results are shown in A radiograph of the abdomen showed a moderate amount of fecal matter in the colon and no dilated bowel loops free air or skeletal abnormality Urinalysis revealed 1 urobilinogen Blood and stool specimens were sent for culture Screening of blood specimens for malaria specific antigens was negative and for cold agglutinins was nonreactive at a dilution of 1 The patient was admitted to the hospital.

**ChatGPT-3.5’s Response:**

Based on the given information, the most likely diagnosis from the provided options is Option I: Ulcerative colitis.

Rationale:

1. Abdominal pain: The patient has persistent upper abdominal pain, which is a common symptom of ulcerative colitis.

2. Pallor: The patient has worsening pallor, which can be associated with chronic gastrointestinal bleeding seen in ulcerative colitis.

3. Diarrhea with blood: The patient has daily loose stools with occasional blood mixed in, which is a characteristic feature of ulcerative colitis.

4. Constitutional symptoms: The patient presents with malaise, fevers, fatigue, and weight loss, which are commonly seen in ulcerative colitis.

5. Elevated pulse rate: Tachycardia (pulse rate of 123 beats per minute) can be a sign of systemic inflammation and dehydration associated with ulcerative colitis.

6. Abdominal tenderness: The patient has tenderness on palpation in the right upper and left lower quadrants of the abdomen, which can be indicative of colonic involvement in ulcerative colitis.

7. Rectal bleeding: The presence of occasional blood mixed in with the loose stools and on the toilet paper suggests rectal involvement in ulcerative colitis.

8. Normal liver function tests: The blood levels of alanine aminotransferase (ALT), alkaline phosphatase, and total and direct bilirubin are normal, indicating that liver disease is less likely as a cause of the symptoms.

9. Normal stool culture: The stool culture showed no occult blood, which suggests that the bloody diarrhea is not due to an infectious cause like Salmonellosis.

10. Normal renal function tests: The results of renal function tests are normal, ruling out renal causes of the patient's symptoms.

Based on these findings, ulcerative colitis is the most likely diagnosis, as it explains the patient's chronic abdominal pain, bloody diarrhea, constitutional symptoms, and laboratory findings. However, further diagnostic evaluations such as colonoscopy and histopathological examination of colonic biopsies would be necessary to confirm the diagnosis.

**GPT4’s Response:**

From the given case and the options, I would consider Salmonellosis (Option IV) as the most likely diagnosis. Here is my rationale:

Salmonellosis is an infection caused by the Salmonella bacteria, commonly due to contaminated food or water. This infection can cause symptoms like abdominal pain, diarrhea, fever, and malaise - all of which are presented in this case. The fact that the boy had traveled to the Dominican Republic, which includes a rural village, three months before his symptoms started suggests a potential exposure to contaminated food or water, a common risk factor for Salmonellosis. The green, loose stools with occasional blood could also be indicative of a Salmonella infection. On the other hand, while symptoms such as abdominal pain, pallor, fever, malaise, weight loss, and fatigue might be consistent with some aspects of Ulcerative colitis (Option I), Hodgkin's lymphoma (Option II), and Polyarteritis nodosa (Option III), there are other typical signs and symptoms of these conditions that are not presented in this case. For instance, Hodgkin's lymphoma often presents with painless enlargement of lymph nodes, night sweats, and unexplained weight loss - none of these symptoms were mentioned in the case. Ulcerative colitis typically has a more chronic course with bloody diarrhea, often accompanied by tenesmus (a constant urge to pass stools) and sometimes joint pains, which aren't described in this case. Lastly, Polyarteritis nodosa, a systemic vasculitis, typically presents with a variety of systemic symptoms that may include fever, fatigue, weight loss, muscle and joint aches, and a rash, and there would be a greater suspicion of this diagnosis if there were signs of organ involvement such as hypertension, mononeuritis multiplex, or abnormal kidney function.

Thus, considering the clinical presentation and the epidemiological context, Salmonellosis would be the most likely diagnosis given the provided options. However, the medical team should still consider other possibilities and conduct further investigations to confirm the diagnosis and to rule out other potential conditions.

**Bard’s Response:**

The most likely diagnosis for this patient is Salmonellosis. The patient has a history of fevers, abdominal pain, and worsening pallor. He also has a history of loose stools with occasional blood mixed in. These are all symptoms of Salmonellosis, which is a bacterial infection that can cause diarrhea, fever, and abdominal cramps. The patient's laboratory tests also support a diagnosis of Salmonellosis. His white blood cell count is elevated, which is a sign of infection. He also has an elevated C-reactive protein level, which is a marker of inflammation. The other possible diagnoses for this patient are Ulcerative colitis, Hodgkin's lymphoma, and Polyarteritis nodosa. However, these diagnoses are less likely because they do not typically cause the same symptoms as Salmonellosis. Ulcerative colitis is an inflammatory bowel disease that causes ulcers in the lining of the colon. It can cause diarrhea, abdominal pain, and rectal bleeding. However, it is less likely to cause fever or pallor. Hodgkin's lymphoma is a cancer of the lymphatic system. It can cause fever, night sweats, weight loss, and fatigue. However, it is less likely to cause abdominal pain or diarrhea. Polyarteritis nodosa is a rare autoimmune disease that causes inflammation of the small blood vessels. It can cause fever, weight loss, and muscle pain. However, it is less likely to cause abdominal pain or diarrhea. In conclusion, the most likely diagnosis for this patient is Salmonellosis. The patient's symptoms, laboratory tests, and travel history all support this diagnosis.

**Example Prompt 3:**

Choose the most likely diagnosis from the following and explain the rationale for your answer: Option I: Visceral Kaposi's sarcoma, Option II: Gastric lymphoma with hemorrhage, Option III: Insulinoma, Option IV: Babesiosis. Feel free to dispute my options. The case is : A 29 year old man was seen in an outpatient clinic affiliated with this hospital because of abdominal pain fever and weight loss The patient was reportedly well until unintentional weight loss occurred approximately 6 weeks before presentation Three weeks before presentation upper abdominal pain developed that the patient rated at 8 on a scale of 0 to 10 with 10 indicating the most severe pain The pain radiated to his throat increased after eating and drinking and did not diminish with ibuprofen At the outpatient clinic the patient reported nausea occasional vomiting decreased food intake and a loss of 10 kg from his usual weight of 4 kg He reported no headaches visual changes neck stiffness dyspnea substernal chest pain lower abdominal pain diarrhea back pain dysuria rashes or joint pain He had had a respiratory illness approximately 2 years before this evaluation He had no known allergies He was born in Central America and had been living in the United States for 4 years He worked in agriculture and did not speak English He smoked cigars occasionally drank alcohol infrequently and did not use illicit drugs On examination the patient appeared cachectic The temperature was 3 C the blood pressure 101 68 mm Hg the pulse 114 beats per minute the respiratory rate 20 breaths per minute and the oxygen saturation 100 while he was breathing ambient air The abdomen was soft with normal bowel sounds and without tenderness or distention There was macular hyperpigmentation of the feet and legs that reportedly had not changed for 10 years The remainder of the examination was normal Urinalysis revealed 1 protein and was otherwise normal Ranitidine was prescribed and the patient returned home with instructions to follow up the next day when test results were available Blood levels of glucose calcium phosphorus lipase and amylase were normal as were the results of tests of liver and renal function Testing for antibodies to hepatitis C virus was negative other test results are shown in A frontal chest radiograph showed subtle tiny nodules throughout both lungs without focal consolidation 1A There was no evidence of mediastinal or hilar lymphadenopathy Two days later the patient returned to the outpatient clinic He reported persistent abdominal pain that he rated at 8 on a scale of 0 to 10 as well as sore throat fever and one episode of hematemesis that had occurred the day before He reported no cough congestion or improvement with ranitidine The temperature was 3 C the blood pressure 105 65 mm Hg the pulse 119 beats per minute the respiratory rate 18 breaths per minute and the oxygen saturation 99 while he was breathing ambient air There were coarse breath sounds at both lung bases The abdomen was firm without distention masses guarding or rebound the remainder of the examination was unchanged Airborne precautions were instituted and a face mask was placed Acetaminophen was administered and a tuberculin skin test was performed The patient was transported to the emergency department of this hospital He was heterosexual and reported having no more than eight partners in his lifetime and consistently using condoms There was no known history of exposure to tuberculosis On examination the temperature was 9 C the blood pressure 100 65 mm Hg the pulse 68 beats per minute the respiratory rate 24 breaths per minute and the oxygen saturation 99 while the patient was breathing ambient air The weight was 6 kg the height 160 cm and the body mass index the weight in kilograms divided by the square of the height in meters A white exudate was present on the underside of the tongue A nontender mobile lymph node 1 to 2 cm in diameter was present in the left submandibular chain and additional nontender mobile lymph nodes were present in the right submental region and the right cervical region Fine rales were scattered throughout both lungs and the abdomen was soft the remainder of the examination was unchanged Blood levels of glucose calcium phosphorus magnesium lipase and amylase were normal as were the results of tests of liver and renal function other test results are shown in Computed tomography CT of the chest performed after the intravenous administration of contrast material revealed numerous nodules 1 to 3 mm in diameter randomly distributed in a miliary pattern i e having an appearance that is similar to millet seeds in both lungs without mediastinal hilar or axillary lymphadenopathy 1B and 1C To help evaluate the patient s abdominal pain a contrast enhanced CT scan of the abdomen and pelvis was obtained that showed multiple enlarged mesenteric lymph nodes and new small bilateral pleural effusions The mesenteric lymph nodes had low attenuation centers that were suggestive of central necrosis 1D There was no evidence of bowel dilatation or bowel wall thickening Specimens of blood sputum and urine were obtained Intravenous saline and one dose of ceftriaxone and azithromycin were administered followed by fluconazole omeprazole and nystatin suspension Results of coagulation tests were normal The patient was admitted to the hospital During the first day the temperature rose to 7 C Acetaminophen was administered.

**ChatGPT-3.5’s Response:**

Based on the provided clinical presentation and diagnostic findings, the most likely diagnosis for this patient appears to be **Option II: Gastric lymphoma with hemorrhage**.

Here's the rationale for this choice:

1. **Clinical Presentation**: The patient presented with several concerning symptoms, including unintentional weight loss, upper abdominal pain, nausea, vomiting, and a sore throat. These symptoms can be associated with various medical conditions, but they are particularly concerning in the context of abdominal pain, weight loss, and a history of fever.

2. **Physical Examination**: The patient's cachectic appearance, low-grade fever, and abnormal lung findings (coarse breath sounds) suggest a systemic illness that may have metastasized or affected multiple organs.

3. **Laboratory Findings**: The presence of a white exudate on the underside of the tongue, cervical lymphadenopathy, and the scattered rales in both lungs are significant clinical findings that point toward lymphatic involvement.

4. **Radiological Imaging**: The chest CT scan showed numerous nodules randomly distributed in a miliary pattern throughout both lungs. This pattern is consistent with lymphatic spread and can be indicative of lymphoma.

5. **Abdominal Imaging**: The abdominal CT scan revealed multiple enlarged mesenteric lymph nodes with central necrosis. These findings are concerning for lymphadenopathy and suggest lymphoma as a possible cause.

6. **Hematemesis**: The patient had an episode of hematemesis, which may be associated with gastrointestinal involvement. Lymphoma can infiltrate the gastrointestinal tract and lead to bleeding.

7. **Demographics**: The patient's age (29 years), origin (Central America), and occupation (agriculture) are less likely to be associated with other options such as Babesiosis or Visceral Kaposi's sarcoma.

While the information provided doesn't rule out other conditions entirely, the combination of clinical symptoms, physical examination findings, and imaging results strongly suggests that gastric lymphoma with hemorrhage is the most likely diagnosis. However, it is essential to conduct further diagnostic tests, including biopsies and histopathology, to confirm this diagnosis and determine the specific subtype of lymphoma involved.

**Bard’s Response:**

The most likely diagnosis is Visceral Kaposi's sarcoma (VKS).

Rationale:

The patient is a young male from Central America, which is a region with a high prevalence of HIV/AIDS. VKS is a common opportunistic infection in HIV-infected patients.

The patient has a classic presentation of VKS, with abdominal pain, fever, weight loss, and lymphadenopathy.

The CT scan of the abdomen and pelvis showed multiple enlarged mesenteric lymph nodes with low attenuation centers, which is suggestive of central necrosis. This is a characteristic finding in VKS.

The patient also has miliary nodules on chest X-ray, which can be seen in VKS.

The other options are less likely:

Gastric lymphoma with hemorrhage: Gastric lymphoma is a rare type of cancer that can cause abdominal pain, fever, weight loss, and bleeding. However, it is more common in older adults and does not typically cause miliary nodules on chest X-ray.

Insulinoma: Insulinoma is a tumor of the pancreas that produces too much insulin. This can lead to low blood sugar, which can cause symptoms such as sweating, tremors, and confusion. However, it is not typically associated with abdominal pain, fever, weight loss, or lymphadenopathy.

Babesiosis: Babesiosis is a tick-borne infection that can cause fever, headache, muscle pain, and fatigue. However, it is rare in the United States and does not typically cause abdominal pain, lymphadenopathy, or miliary nodules on chest X-ray.

Given the patient's presentation and the findings on imaging, VKS is the most likely diagnosis.

**GPT4’s Response:**

Option I: Visceral Kaposi's Sarcoma

- Kaposi's sarcoma is a cancer caused by Human Herpesvirus 8 (HHV-8). It commonly affects the skin, but can also affect internal organs.

- The patient’s symptoms of abdominal pain, fever, weight loss, and cachexia could be consistent with this diagnosis.

- The patient’s macular hyperpigmentation of the feet and legs could be a manifestation of Kaposi’s sarcoma on the skin.

- However, the CT findings of miliary nodules in the lungs, enlarged mesenteric lymph nodes, and pleural effusions are not typical of Kaposi’s sarcoma.

Option II: Gastric Lymphoma with Hemorrhage

- Gastric lymphoma can present with abdominal pain, weight loss, and nausea/vomiting.

- The patient's one episode of hematemesis could suggest gastrointestinal bleeding, potentially from the lymphoma.

- However, the patient's respiratory symptoms, lung nodules, and lymphadenopathy are not typical of gastric lymphoma.

Option III: Insulinoma

- Insulinoma is a rare pancreatic tumor that secretes insulin, leading to hypoglycemia.

- The patient’s normal blood glucose level makes this diagnosis unlikely.

- The patient's symptoms and imaging findings do not align with an insulinoma.

Option IV: Babesiosis

- Babesiosis is a parasitic infection that can be transmitted through tick bites, which may be relevant given the patient’s work in agriculture.

- Symptoms can include fever, chills, sweats, malaise, fatigue, and weight loss.

- The lung nodules and enlarged mesenteric lymph nodes could potentially be manifestations of disseminated babesiosis, but this is less common.

- Further testing (e.g., blood smears, PCR for Babesia) would be required to confirm this diagnosis.

Given the limited information available, none of the provided options perfectly fits the patient’s presentation. However, the patient’s symptoms, imaging findings, and history of living in Central America and working in agriculture raise the possibility of an infectious or parasitic disease. Babesiosis or another tick-borne illness could be considered, though further testing would be required to confirm this. Additionally, other infectious diseases endemic to Central America, such as tuberculosis or fungal infections, should be considered and ruled out. Tuberculosis, in particular, could explain the lung nodules, enlarged lymph nodes, and the patient’s systemic symptoms, making it a strong differential diagnosis to consider. Further testing, including sputum cultures, acid-fast bacillus (AFB) smears, and PCR for Mycobacterium tuberculosis, would be needed to investigate this possibility.

There appears to be a typographical error in the temperature recordings, as a temperature of 3 C (37.4 F) would be incompatible with life. This needs clarification for a more accurate assessment. Additionally, the weight loss from 4 kg to 6 kg seems to be a typographical error as well, as these weights are below normal for an adult, and the context suggests significant weight loss rather than gain.

It’s also worth noting that the patient's condition appears to be worsening, as indicated by the increasing temperature and the development of new symptoms. This urgency underscores the need for rapid diagnostic work-up and appropriate management.

**MedAlpaca’s Response:**
Answer: 1A is Kaposi's sarcoma.

The appearance of the chest x-ray is not typical of lymphoma.

The CT scan of the abdomen and pelvis is highly indicative of AIDS-related Kaposi's sarcoma.

The appearance of the CT scan is highly indicative of Kaposi's sarcoma.
